# Supplementary material for: Tigecycline Sensitivity Reduction in Escherichia coli Due to Widely Distributed tet(A) Variants
Source: Microorganisms. 2023 Dec 18;11(12):3000. doi: 10.3390/microorganisms11123000 (PMC10745318; doi:10.3390/microorganisms11123000)
Supplement: Supplementary file 1 [file microorganisms-11-03000-s001.zip › Supplementary Data/Supplementary Data_last_vision.pdf]

Supplementary information

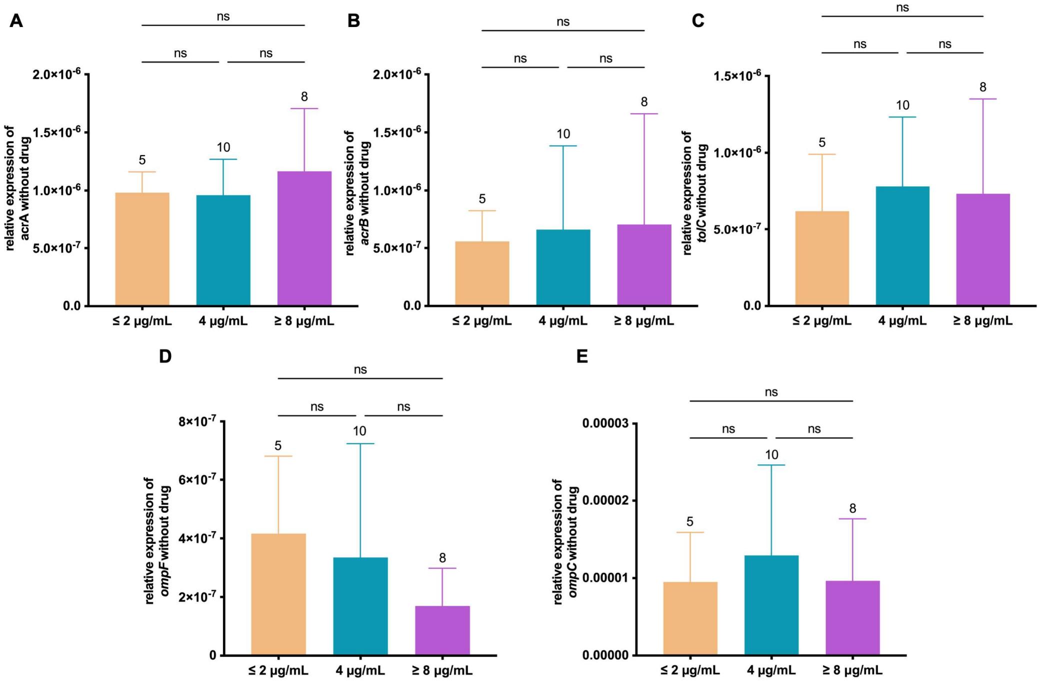

Supplementary Figure S1: The relative expression of *acrA* (A), *acrB* (B), *tolC* (C), *ompF* (D) and *ompC* (E) without tigecycline.

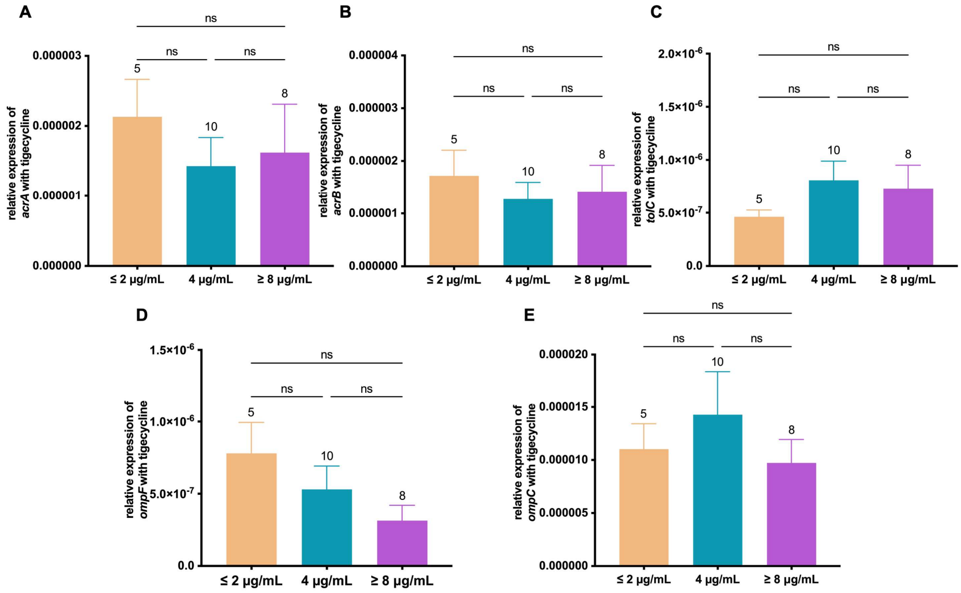

Supplementary Figure S2: The relative expression of *acrA* (A), *acrB* (B), *tolC* (C), *ompF* (D) and *ompC* (E) with 0.5 μg/mL tigecycline.

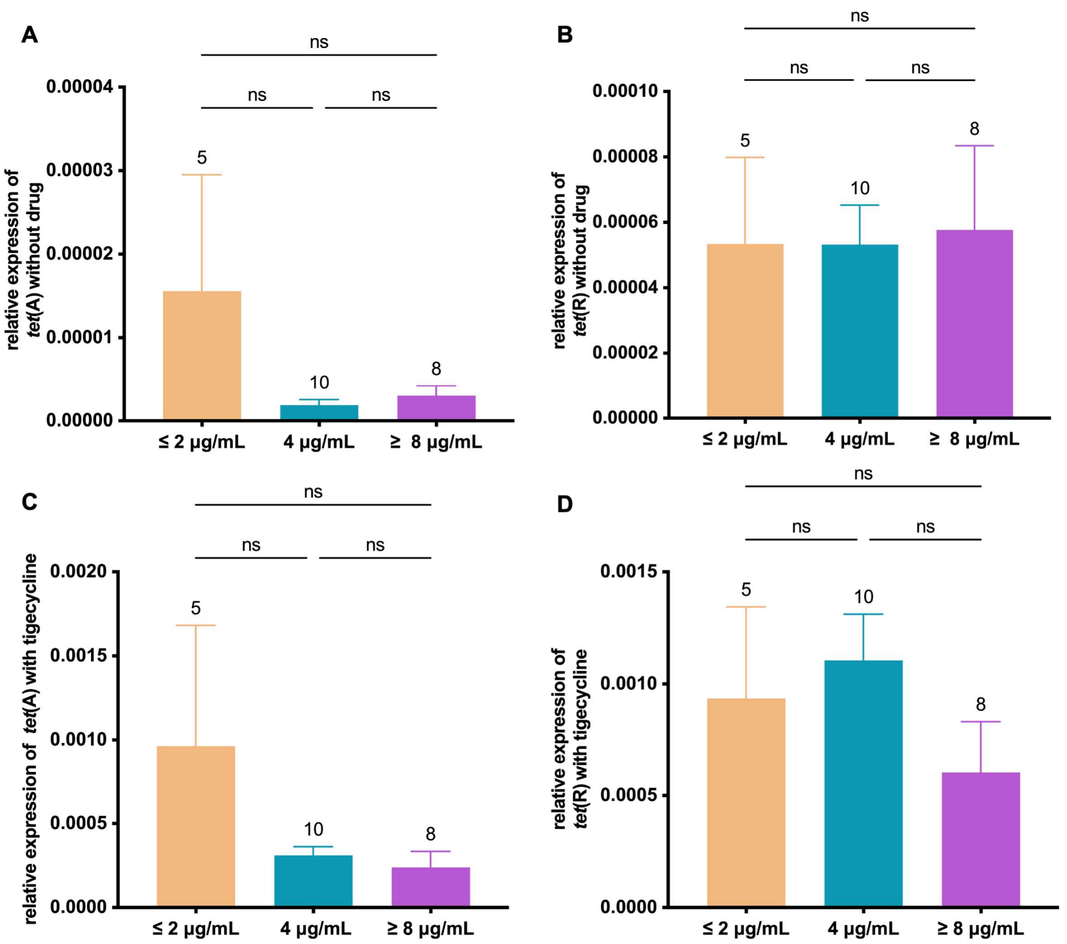

Supplementary Figure S3: The relative expression of *tet*(A) and *tet*(R) without (A, B) or with 0.5 μg/mL (C, D) tigecycline.

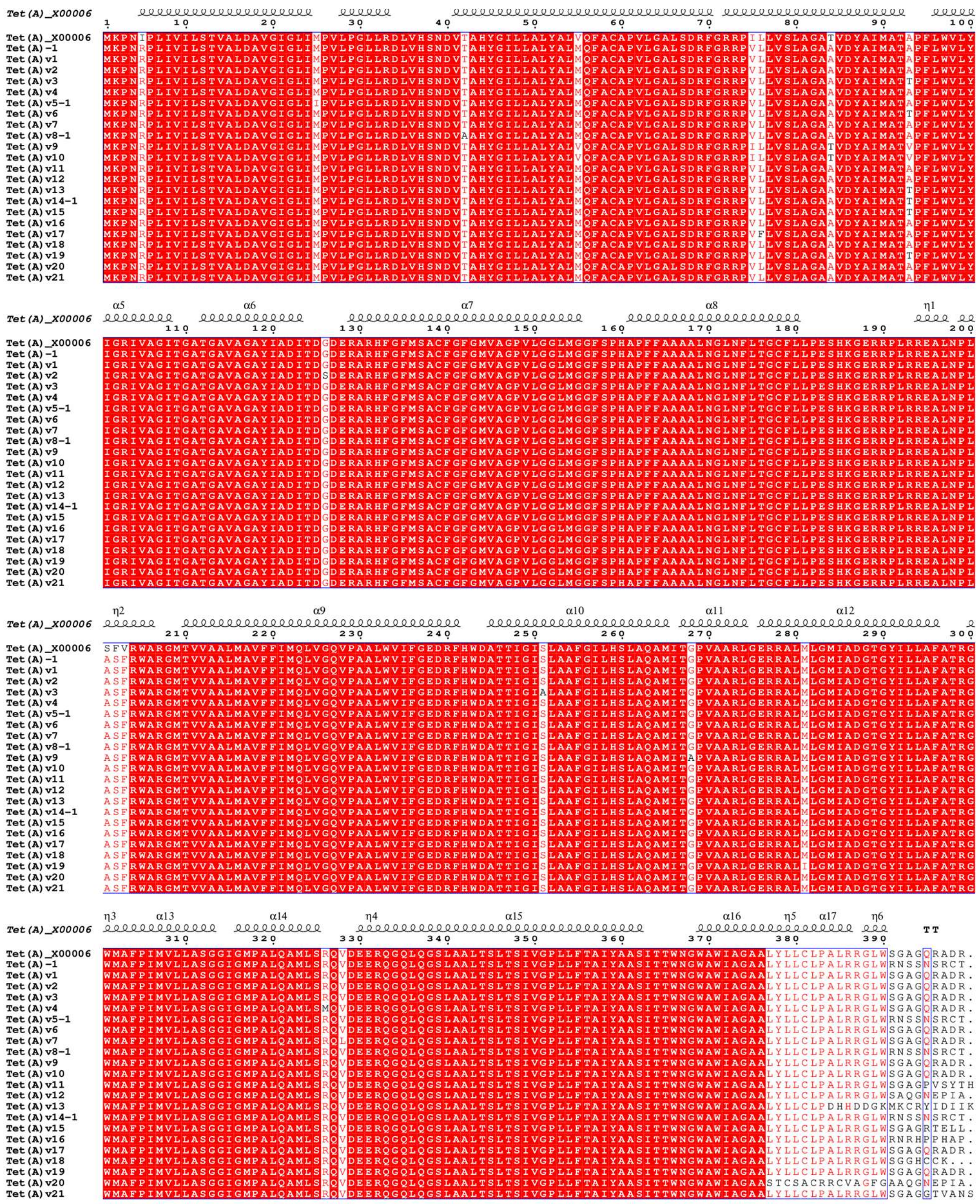

Supplementary Figure S4: Amino acid differences of 21 *tet*(A) variants from the world.

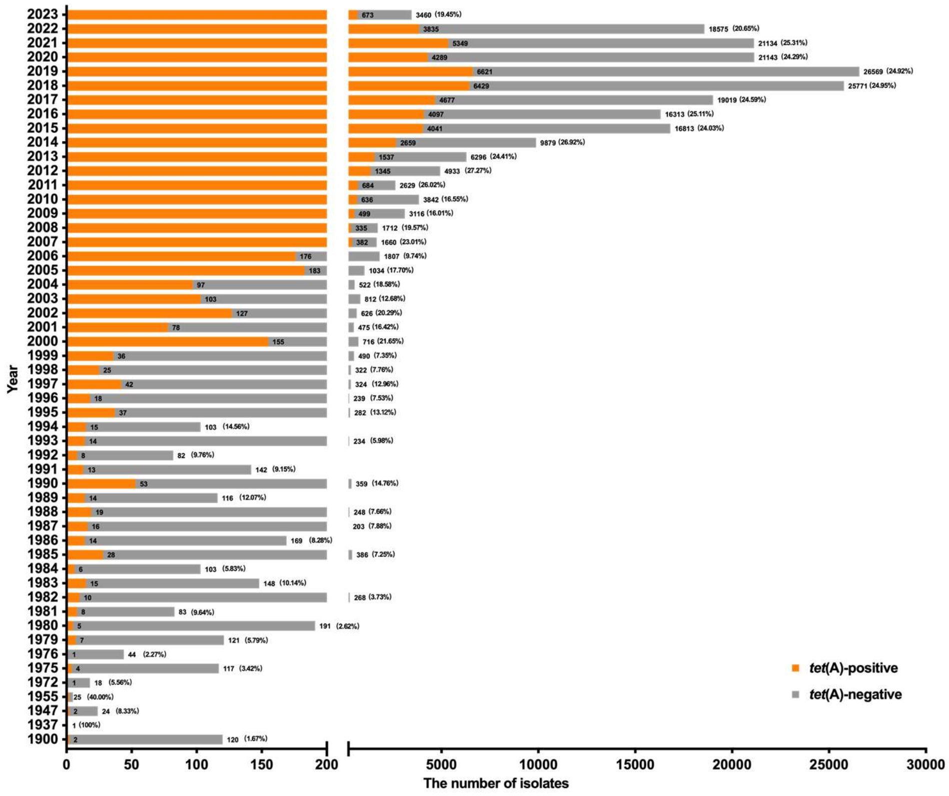

Supplementary Figure S5: The presence of all *tet*(A)-variant-harbouring *E. coli* from the whole world in different years.

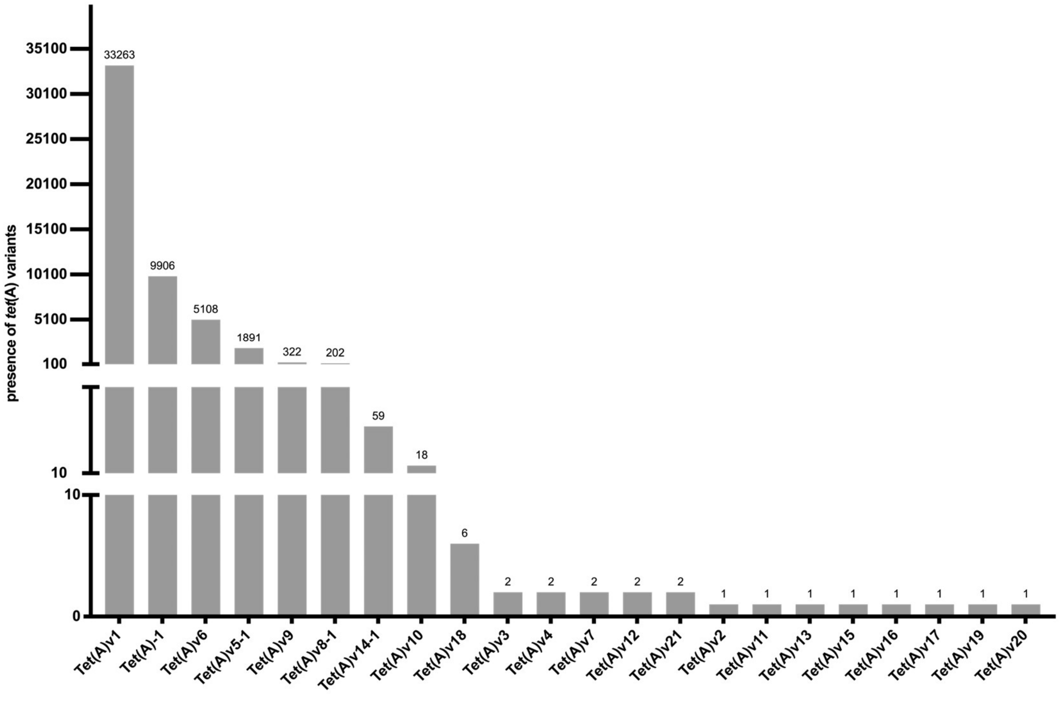

Supplementary Figure S6: The prensence of different *tet*(A)-variant-harbouring *E. coli* from the world.

Table S1 The primers for cloning all *tet*(A) variants

Primer name	Primers (5'-3')
*tet*A-*tet*R-insert-F	aaaacgacggccagtgaattGCTTGCCGGAAGTCGCCT
*tet*A-*tet*R-insert-R	gaccatgattacgccaagctCTCTGCTGTAGTGAGTGGGTTGC
pUC19-linear-F	AGCTTGGCGTAATCATGGTCATAG
pUC19-linear-R	AATTCACTGGCCGTCGTTTTACAA

Table S2 The primers for point mutation

Primer name	Primers (5'-3')
pUC19_I5R_F	gttgggtttcacgtctggcc
pUC19_I5R_R	tcgtggaaacgataggcctatg
mutant_I5R_F	taggcctatcgtttccacgaTCAGCGATCGGCTCGTTG
mutant_I5R_R	ggccagacgtgaaacccaacAGACCCCTGATCGTAATTCTGAGC
pUC19_V55M_F	caacgcatacagcgccagc
pUC19_V55M_R	tacagacaagctgtgaccgtctcc
mutant_V55M_F	acggtcacagcttgtctgtaAGCGGATGCCGGGAGCAG
mutant_V55M _R	tgctggcgctgtatgcgttgATGCAATTTGCCTGCGCA
pUC19_I75V_F	tggccgccgcccgaaacg
pUC19_ I75V _R	tacagacaagctgtgaccgtctcc
mutant_I75V_R	atcgtttcgggcggcggccgGTCTTGCTCGTCTCGCTGGC
mutant_I75V_F	acggtcacagcttgtctgtaAGCGGATGCCGGGAGCAG
pUC19_T84A_F	ggcgccggccagcgagac
pUC19_T84A_R	tacagacaagctgtgaccgtctcc
mutant_T84A_F	acggtcacagcttgtctgtaAGCGGATGCCGGGAGCAG
mutant_T84A_R	tcgtctcgctggccggcgctGCTGTCGACTACGCCATCATGG
pUC19_ASF_F	gagcgggttgagagcctcc
pUC19_ASF_R	ttcaccgtcatcaccgaaacg
mutant_ASF_F	gtttcggtgatgacggtgaaAACCTCTGACACATGCAGCTCC
mutant_ASF_R	gggaggctctcaacccgctcGCTTCGTTcCGGTGGGCCCGGGGCAT

Table S3 The primer for qPCR

Genes	Primers (5'-3')	Standard curve	Lengh (bp)	Annealing temp (℃)	Reference
acrB	F: 5′-CAAGGAAACGAACGCAATACC-3′<br>R: 5′-AGTCGGTGTTCGCCGTTAAC-3′	Ct = -3.170 logcopies + 38.541	74	60	[32]
acrA	F: 5′-TGCAGAGGTTCAGTTTTGACTGTT-3′<br>R: 5′-CTCTCAGGCAGCTTAGCCCTAA-3′	Ct = -3.134 logcopies + 38.040	107	60	[32]
ompF	F: 5′-CGTACTTCAGACCAGTAGCC-3′<br>R: 5′-GAACTTCGCTGTTCAGTACC-3′	Ct = -3.2 logcopies + 37.897	209	60	[33]
ompC	F: 5′-ATTCTGGCAGTACGTCGGTC-3′<br>R: 5′-AAACAACTCCTGGACCCGTG	Ct = - 3.087 logcopies + 37.298	125	60	[34]
tolC	F: 5′-AAGCCGAAAAACGCAACCT-3′<br>R: 5′-CAGAGTCGGTAAGTGACCATC-3′	Ct = - 3.05 blogcopies + 37.106	101	60	[35]
